# Synthesis, Spectroscopic, and Antimicrobial Studies of Binuclear Metallocene (M = Ti, Zr, or Hf) Derivatives of Bis(mercaptoazoles)

**DOI:** 10.1155/2007/87918

**Published:** 2007-05-22

**Authors:** Shilpi Sinha, Akhilesh Kumar Srivastava, Chandra Mohan Tripathi, Om Prakash Pandey, Soumitra Kumar Sengupta

**Affiliations:** Department of Chemistry, DDU Gorakhpur University, Gorakhpur 273009, India

## Abstract

The reactions of (*η* ^5^ − C_5_H_5_)_2_MCl_2_ (M = Ti, Zr, or Hf) with mercaptoazoles (LH_2_), namely, bis(mercaptotriazoles), bis(mercap- tooxadiazoles), and bis(mercaptothiadiazoles) in 2 : 1 molar ratio, respectively, have been studied in dry tetrahydrofuran in the presence of *n*-butylamine and the binuclear complexes of the type [{(*η* − C_5_H_5_)_2_ M}_2_(L)] (M = Ti/Zr/Hf) are obtained. Tentative structural conclusions are drawn for the reaction products based upon elemental analysis, electrical conductance, magnetic moment, and spectral data (UV-Vis, IR, ^1^H NMR, and ^13^C NMR). FAB-mass spectra of few complexes of each series were also carried out to confirm the binuclear structures. Studies were conducted to assess the growth-inhibiting potential of the complexes synthesized, and the ligands against various fungal and bacterial strains.

## 1. INTRODUCTION

The chemistry of transition metal complexes containing heterocyclic thione donors continues to be of interest on account of their interesting structural features and also because of their biological importance [1–6]. The combination of the exocyclic thione/thiol group and the heterocyclic molecule, which may contain nitrogen, oxygen, or sulphur or a combination thereof, generates a group of molecules with considerable coordination potential [1–3]. The coordination behavior of such molecule depends upon reaction conditions, nature of metal ion, and pH of the medium. The stimulus for much of the research into the coordination chemistry of heterocyclic thione/thiol donors status from their wide ranging applications [7–9], *viz.*, in analytical chemistry, in metal finishing, and electroplating industries uses as polyolefin stabilizers and as vulcanization accelerators. Fungicidal, insecticidal, and acaricidal activities have also been reported. Other biological applications include thyrotoxic activity; centred nervous system depressant and a platinum pyridine thione complex have been patented for clinical use in cancer treatment [1–3]. However, so far no report is available on the coordination behavior of bis(mercaptoazoles).

**Scheme 1:**
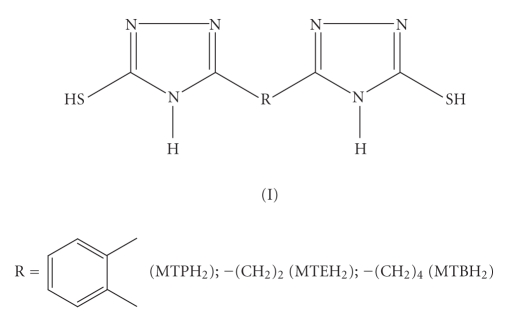
Bis(mercaptotriazoles).

**Scheme 2:**
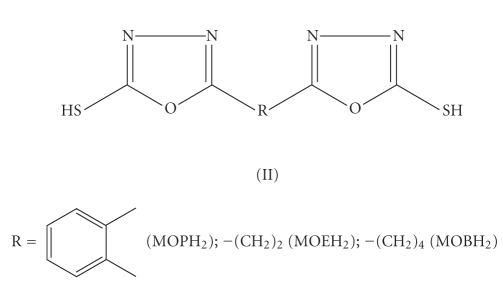
Bis(mercaptooxadiazoles).

**Scheme 3:**
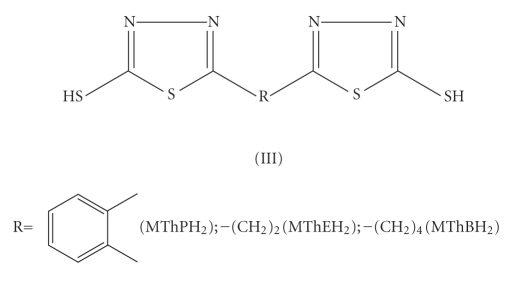
Bis(mercaptothiadiazoles).

In this paper, we describe the synthesis, characterization, antifungal, and antibacterial studies on titanium(IV)/zirconium(IV)/hafnium(IV) derivatives containing three im-portant series of bis(mercaptoazoles) *viz.*, bis(mercaptotriazoles) (I), bis(mercaptooxadiazoles) (II), and bis(mercaptothiadiazoles) (III) as coligands alongwith cyclopentadienyl group. The structures of ligands, used for the present study, are shown in Schemes 1, 2, and 3.

## 2. EXPERIMENTAL

All manipulations were performed under anhydrous conditions under a dry O_2_ free N_2_ atmosphere. Extreme precautions were taken to exclude moisture. Tetrahydrofuran was dried by distilling it over sodiumwire or pieces. Bis(cyclopentadienyl)titanium(IV)/zirconium(IV)/hafnium(IV) dichloride was purchased from Aldrich chemical Co. (Wis, USA) The ligands were prepared as reported in the literature [10]. The details of analytical methods and physical measurements were the same described earlier [11].

The antibacterial activity was evaluated by the paper-disc plate method [12]. The nutrient agaz medium (peptone, NaCl, and agar) and 5mm diameter paper discs of Whatman No. 1 were used. The filter paper discs were soaked in different solutions of the compounds, dried and then placed in the petriplates previously seeded with the test organism (Gram-positive *Bacillus subtilis* and Gram-negative *Escherichia coli*). The plates were incubated for 24 hours at 30 ± 1°C and the inhibition around each disc was measured in mm.

### 2.1. Preparation of complexes

To a solution of bis(cyclopentadienyl)titanium(IV)/zirconium(IV)/hafnium(IV), chloride (20 mmol) in dry tetrahydrofuran (∼40 cm^3^) was added appropriate bis(mercapto-triazole/oxadiazole/thiadiazole) (10 mmol). To the resulting solution, dry *n*-butylamine (20 mmol) was added and the mixture was stirred for several hours at room temperature. *n*-butylamine hydrochloride remains soluble in tetrahydrofuran. The colored precipitate, thus obtained, was thoroughly washed with tetrahydrofuran and dried in *vacuo*.

For the sake of brevity, the details of all reactions along with physical characteristics and analytical data of the products are given in Table 1.

## 3. RESULTS AND DISCUSSION

Bis(cyclopentadienyl)titanium(IV)/zirconium(IV)/hafnium (IV) chloride reacts with bis(mercaptoazoles) (LH_2_) *viz.*, bis(mercaptotriazoles), bis(mercaptooxadiazoles), or bis(mercaptothiadiazoles) in 2 : 1 molar ratio, respectively, in dry tetrahydrofuran in the presence of *n*-butylamine to give binuclear products of type [{(*η* ^5^ -C_5_H_5_)_2_MCl}_2_(L)], according to the following equation:

(1) $$\begin{array}{l} {}[(\eta^{5}-{\text{C}}_{5}{\text{H}}_{5}{)}_{2}\text{MC}{\text{l}}_{2}]+\text{L}{\text{H}}_{2}+n-2\text{BuN}{\text{H}}_{2}\\ \;\;\overset{\;\text{THF}\;}{\to }[\{(\eta^{5}-{\text{C}}_{5}{\text{H}}_{5}{)}_{2}\text{MCl}{\}}_{2}(\text{L})]+2n-\text{Bu}\text{N}{\text{H}}_{2}\cdot \text{H}\text{Cl}. \end{array}$$

LH_2_ is equal to 1,2-bis(5-mercapto-1,3,4-triazole-2-yl)phenyl (MTPH_2_); 1,2-bis(5-mercapto-1,3,4-triazole-2-yl)ethane (MTEH_2_); 1,4-bis(5-mercapto-1,3,4-triazole-2-yl)butane (MTBH_2_); 1,2-bis(5-mercapto-1,3,4-oxadiazole-2-yl)phenyl (MOPH_2_); 1,2-bis(5-mercapto-1,3,4-oxadiazole-2-yl)ethane (MOEH_2_); 1,4-bis(5-mercapto-1,3,4-oxadiazole-2-yl)butane (MOBH_2_); 1,2-bis(5-mercapto-1,3,4-thiodiazole-2-yl)phenyl (MThPH_2_); 1,2-bis(5-mercapto-1,3,4-thiadiazole-2-yl) ethane (MThEH_2_); 1,4-bis(5-mercapto-1,3,4-Thiadiazole-2-yl)butane (MThBH_2_).

The physical properties and the analytical data of the complexes are given in Table 1. The molecular weights of few complexes, as obtained from ion peak in FAB-mass spectra, are also given in Table 1. The complexes are colored solids and are soluble in dimethylformamide and dimethyl sulphoxide. These complexes have high decomposition temperature and do not decompose up to 250°C. The electrical conductance measurements in dimethylformamide are consistent with their nonelectrolytic nature. Magnetic susceptibility values at room temperature show their diamagnetic nature.

### 3.1. Electronic spectra

The electronic spectra of complexes, recorded in dimethylformamide, show a single band in the region 22 800–24 000cm^−1^ which can be assigned [13] to the charge-transfer bond. In addition, the ligand and the complexes show band around 32 000cm , which is assigned to *π* → π* transition of the azomethine linkage.

### 3.2. Infrared spectra

The important infrared spectra of the ligands, mercapto azoles, and their corresponding titanium(IV)/zirconium(IV)/hafnium(IV) derivatives are given in Table 2. The assignments of i.r. spectral ligand bands and the complexes are based on earlier studies of similar ligand [14–18]. All complexes show bands at *ca.* 3000 cm^−1^ , 1420 cm^−1^ , and 1020 cm^−1^ indicating the presence of cyclopentadienyl ring attached to titanium(IV)/zirconium(IV)/hafnium(IV) ion. All these bonds are similar to those reported [19] for bis(cyclopentadienyl)titanium(IV)/zirconium(IV)/hafnium (IV) chloride. The appearance of these bands for cyclopentadienyl ring indicates that (*η* ^5^ -C_5_H_5_) group remains in the complexes. The infrared spectra of bis(mercaptotriazoles), bis(mercaptooxadiazoles), and bis(mercaptothiadiazoles) show one weak band at 2480–2550 cm^−1^ due to the −SH group vibration. However, in the spectra of complexes, this band disappears indicating the coordination through sulphur after deprotonation. This is further supported [11] by the appearance of band at *ca.* 340–380 cm^−1^, assignable to *ν* (M−S). A strong band in the region of 1585–1560 cm^−1^ in the ligands is characteristics [18] of *ν*(C=N) ring group. However, in the complexes the *ν*(C=N) band is found to split in two; where one band is located almost at the original position, that is, at *ca.* 1580 cm^−1^ due to uncoordinated *ν*(C=N) and other is shifted to lower frequency (∼ 20–25cm^−1^) arising from the coordinated (C=N) mode. The splitting of *ν* (C=N) absorption band suggests that only one nitrogen from each unit of mercaptotriazole, mercaptooxadiazole, and mercaptothiadiazole is involved in coordination. The bands observed at 410–440 cm^−1^ may be assigned to *ν* (M−N). The infrared spectra of bis(mercaptotriazoles) show one band at 3150 cm^−1^ assignable [20] to *ν* (N−H). The bands due to *ν* (C−O−C) in bis(mercaptooxadiazoles) appear at *ca.* 1290 cm^−1^ (symmetric) and 1350 cm^−1^ (asymmetric); while bis(mercaptothiadiazoles) show band at *ca.* 660–650 due to *ν*(C−S). The position of infrared bands due to phenyl and heterocyclic (triazole, oxadiazole, or thiadiazole) ring does not change in the complexes indicating the noncoordination of nitrogen (triazole ring), oxygen (oxadiazole ring), or sulphur (thiadiazole ring) atoms.

Thus, the infrared spectra reflect that all bis(mercaptoazoles), that is, bis(mercaptotriazoles), bis(mercaptooxadiazoles), and bis(mercaptothiadiazoles) act as dibasic, tetradentate chelating agents coordinating through two thiol sulphur atoms and two ring azomethine nitrogen atoms.

**^1^H NMR spectra**

The proton magnetic resonance spectra of ligands and their corresponding bis(cyclopentadienyl)titanium(IV)/zirconium(IV)/hafnium(IV) derivatives were recorded (Table 3) in DMSO–d_6_. The intensities of all the resonance lines were determined by planimetric integration. The following conclusions can be derived by comparing the spectra of ligands and their corresponding derivatives.

- The signal due to –SH proton appears at ca. *δ* 8.8–9.0 in the ligands which disappears in the corresponding bis(cyclopentadienyl)titanium(IV)/zirconium(IV)/hafnium(IV) derivatives.
- A signal in all the derivatives at *δ* 6.58–6.72 may be assigned to the protons of the cyclopentadienyl rings. The appearance of single, sharp signal for cyclopentadienyl ring indicates that there is rapid rotation of the cyclopentadienyl ring around the metal ring axis.

**^13^C NMR spectra**

The ^13^C NMR spectra of ligands and the corresponding complexes were recorded in DMSO. The ^13^C resonance signals are assigned according to chemical shift theory. The C_5_H_5_ rings give rise to one resonance at ca. *δ* 115.0. The considerable shift in the position of carbons (attached with mercapto group in the ligands; *δ* 150–160) indicates the coordination through mercapto group.

Thus, on the basis of elemental analysis, electrical conductance and spectral data, the following structures (IV) are tentatively proposed for titanium(IV)/zirconium(IV)/ hafnium(IV) complexes. Proposed binuclear structure has also been confirmed by FAB mass spectra of few complexes of each series.

Attempts are being made to grow single crystal of the complexes suitable for X-ray studies but so far no success has been achieved.

### 3.3. Antifungal activity

The fungicidal activity of the ligands and their corresponding complexes were evaluated in DMF against *Aspergillus niger, Aspergillus fumigate, and Helminothosporim oryzae* by the agar plate technique at 1000, 100, and 10 ppm concentration with triplicate determination in each case. The average percentage inhibition was calculated using the expression: (%) = 100(C−T)/C where C and T are the diameters of the fungus colony in control and test plates, respectively. The recorded results (Table 4) lead to the following conclusions.

- The compounds show significant toxicity at 1000ppm concentration against all species of fungi. However, the complexes are more toxic than ligands, which may be owing to the chelation and the presence of sulphur atom.
- The activity decreases on detection.
- Titanium complexes show better activity than zirconium and hafnium complexes. Zirconium and hafnium complexes show almost similar results. This may be due to similar radius of zirconium and hafnium.
- For a particular metal, the complexes with bis(mercaptothiadiazoles) show better activity.
- For a particular series of ligands, the compounds with R = C_6_H_4_ show better activity as compared to R = −CH_2_−CH_2_− or −(CH_2_)_4_.

The variation in the effectiveness of different biocidal agents against different organisms [21] depends upon the permeability of the cells or differences in ribosomes of antimicrobial agent.

### 3.4. Antibacterial activity

The antibacterial activity of the complexes together with the parent ligands has been screened against Gram-positive *Bascillus subtilis* and Gram-negative *Eschericlia coli* at 1000pm concentration. The results (Table 5) show that activity increases on chelation. The activity of the ligands is affected by the nature of substituents; this in relation to the lipophilicity of the ligands and their membrane permeability, a key factor in determining the entry inside the cell. The results lead to the following conclusions.

- The complexes are slightly more toxic than the parent ligands.
- The titanium complexes show better activity than zirconium and hafnium complexes.
- The ligands bis(mercaptothiadiazoles) and their complexes show slightly better activity than bis(mercaptotriazoles) and their derivatives which in turn show slightly better activity than bis(mercaptooxadiazoles) and their derivatives.
- The presence of phenyl ring at R increases the antibacterial activity.

## Figures and Tables

**Scheme 4:**
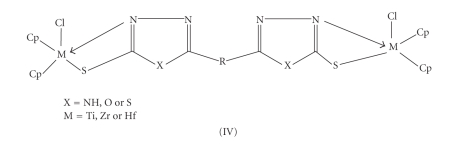


**Table 1:** Reactions of Cp_2_MCl_2_ (M = Ti/Zr/Hf) with bis(mercaptoazoles).

Reactants taken Molar ratio	Stirr. time (hrs)	Product, color, yield (%)	Mol. Wt. Calcd. (found)	Calcd. (found) %					
				C	H	N	S	Cl	M
Cp_2_TiCl_2_ + MTPH_2_ + *n*BuNH_2_	25	[{Cp_2_TiCl}_2_MTP]	701.36	55.6	3.9	10.8	8.2	9.1	12.3
(2 : 1 : 2)		Orange, 68	(701)	(55.5)	(3.5)	(10.6)	(8.1)	(8.9)	(12.1)
Cp_2_ZrCl_2_ + MTPH_2_ + *n*-BuNH_2_	27	[{Cp_2_ZrCl_2_MTP}]	788.04	50.0	3.5	9.7	7.4	8.2	21.1
(2 : 1 : 2)		Yellow, 66	(788)	(50.0)	(3.2)	(9.5)	(7.2)	(8.0)	(21.0)
Cp_2_HfCl_2_ + MTPH_2_ + *n*-BuNH_2_	25	[{Cp_2_HfCl}_2_MTP]	962.58	37.4	2.7	8.7	6.7	7.4	37.1
(2 : 1 : 2)		Light Orange, 68	(963)	(37.2)	(2.6)	(8.5)	(6.5)	(7.2)	(37.0)
Cp_2_TiCl_2_ + MTEH_2_ + *n*-BuNH_2_	23	[{Cp_2_TiCl}_2_MTE]	—	47.8	4.0	12.9	9.8	10.9	14.7
(2 : 1 : 2)		Dark Brown, 67		(47.7)	(3.8)	(12.6)	(9.6)	(10.6)	(14.2)
Cp_2_ZrCl_2_ + MTEH_2_ + *n*-BuNH_2_	20	[{Cp_2_ZrCl}_2_MTE]	—	42.2	3.5	11.4	8.7	9.6	24.7
(2 : 1 : 2)		Cream, 65		(42.1)	(3.2)	(11.1)	(8.4)	(9.2)	(24.3)
Cp_2_HfCl_2_ + MTEH_2_ + *n*-BuNH_2_	24	[{Cp_2_HfCl}_2_MTE]	—	34.1	2.9	9.2	7.0	7.7	39.0
(2 : 1 : 2)		Brown, 60		(34.0)	(2.8)	(9.0)	(7.0)	(7.3)	(39.0)
Cp_2_TiCl_2_ + MTBH_2_ + *n*-BuNH_2_	23	[{Cp_2_TiCl}_2_MTB]	—	49.3	4.4	12.3	9.4	10.4	14.0
(2 : 1 : 2)		Dark Brown, 62		(49.1)	(4.2)	(12.1)	(9.2)	(10.1)	(14.0)
Cp_2_ZrCl_2_ + MTBH_2_ + *n*-BuNH_2_	21	[{Cp_2_TZrCl}_2_MTB]	—	43.8	3.9	10.9	8.3	9.2	23.8
(2 : 1 : 2)		Yellow, 65		(43.7)	(3.6)	(10.7)	(8.2)	(9.0)	(23.4)
Cp_2_HfCl_2_ + MTBH_2_ + *n*-BuNH_2_	20	[{Cp_2_HfCl}_2_MTB]	—	35.7	3.2	8.9	6.8	7.5	37.9
(2 : 1 : 2)		Yellow, 67		(35.5)	(3.0)	(8.8)	(6.6)	(7.4)	(37.8)
Cp_2_TiCl_2_ + MOPH_2_ + *n*-BuNH_2_	20	[{Cp_2_TiCl}_2_MOP]	—	55.5	3.6	7.2	8.2	9.1	12.3
(2 : 1 : 2)		Yellow, 68		(55.3)	(3.5)	(7.0)	(8.0)	(9.0)	(12.0)
Cp_2_ZrCl_2_ + MOPH_2_ + *n*-BuNH_2_	22	[{Cp_2_ZrCl}_2_MOP]	—	49.9	3.3	6.5	7.4	8.2	21.0
(2 : 1 : 2)		Brown, 65		(49.6)	(3.0)	(6.2)	(7.2)	(8.0)	(20.8)
Cp_2_HfCl_2_ + MOPH_2_+ *n*-BuNH_2_	27	[{Cp_2_HfCl}_2_MOP]	—	37.4	2.5	5.8	6.6	7.3	37.0
(2 : 1 : 2)		Light Brown, 65		(37.2)	(2.5)	(5.7)	(6.5)	(7.2)	(36.8)
Cp_2_TiCl_2_ + MOEH_2_ + *n*-BuNH_2_	21	[{Cp_2_TiCl}_2_MOE]	655.28	47.7	3.7	8.6	9.8	10.8	14.6
(2 : 1 : 2)		Yellowish Brown, 60	(655)	(47.3)	(3.3)	(8.4)	(9.6)	(10.6)	(14.3)
Cp_2_ZrCl_2_ + MOEH_2_ + *n*-BuNH_2_	24	[{Cp_2_ZrCl}_2_MOE]	741.96	42.1	3.3	7.6	8.6	9.6	24.6
(2 : 1 : 2)		Dark Brown, 57	(742)	(42.0)	(3.0)	(7.4)	(8.4)	(9.2)	(24.2)
Cp_2_HfCl_2_ + MOEH_2_ + *n*-BuNH_2_	23	[{Cp_2_HfCl}_2_MOE]	916.50	34.1	2.6	6.1	7.0	7.7	38.9
(2 : 1 : 2)		Brown, 62	(916)	(34.0)	(2.3)	(6.0)	(6.8)	(7.5)	(38.5)
Cp_2_TiCl_2_ + MOBH_2_ + *n*-BuNH_2_	25	[{Cp_2_TiCl}_2_MOB]	—	49.2	4.1	8.2	9.4	10.4	14.0
(2 : 1 : 2)		Yellow, 62		(49.0)	(4.0)	(8.0)	(9.2)	(10.1)	(14.0)
Cp_2_ZrCl_2_ + MOBH_2_ + *n*-BuNH_2_	25	[{Cp_2_ZrCl}_2_MOB]	—	43.7	3.7	7.3	8.3	9.2	23.7
(2 : 1 : 2)		Cream, 60		(43.6)	(3.6)	(7.1)	(8.0)	(9.0)	(23.4)
Cp_2_HfCl_2_ + MOBH_2_ + *n*-BuNH_2_	29	[{Cp_2_HfCl}_2_MOB]	—	35.6	3.0	5.9	6.8	7.5	37.8
(2 : 1 : 2)		Brown, 65		(35.4)	(3.0)	(5.3)	(6.5)	(7.4)	(37.6)
Cp_2_TiCl_2_ + MThPH_2_ + *n*-BuNH_2_	20	[{Cp_2_TiCl}_2_MThP]	—	53.2	3.5	6.9	15.8	8.7	11.8
(2 : 1 : 2)		Yellow, 68		(53.0)	(3.3)	(6.7)	(15.7)	(8.5)	(11.5)
Cp_2_ZrCl_2_ + MThPH_2_ + *n*-BuNH_2_	23	[{Cp_2_ZrCl}_2_MThP]	—	48.1	3.1	6.2	14.3	7.9	20.3
(2 : 1 : 2)		Cream, 65		(48.0)	(3.0)	(6.0)	(14.1)	(7.7)	(20.1)
Cp_2_HfCl_2_ + MThPH_2_ + *n*-BuNH_2_	22	[{Cp_2_HfCl}_2_MThP]	—	36.1	2.4	5.6	12.9	7.1	35.8
(2 : 1 : 2)	Dark Brown, 65	(36.0)		(2.2)	(5.5)	(12.8)	(7.0)	(35.6)
Cp_2_TiCl_2_ + MThEH_2_ + *n*-BuNH_2_	18	[{Cp_2_TiCl}_2_MThE]	—	45.4	3.5	8.2	18.7	10.8	13.9
(2 : 1 : 2)		Yellow, 65		(45.2)	(3.3)	(8.0)	(18.4)	(10.1)	(13.7)
Cp_2_ZrCl_2_ + MThEH_2_ + *n*-BuNH_2_	20	[{Cp_2_ZrCl}_2_MThE]	—	40.3	3.1	7.2	16.6	9.2	23.6
(2 : 1 : 2)		Yellowish Brown, 62		(40.1)	(3.0)	(7.0)	(16.3)	(9.0)	(23.2)
Cp_2_HfCl_2_ + MThEH_2_ + *n*-BuNH_2_	26	[{Cp_2_HfCl}_2_MThE]	—	32.9	2.5	5.9	13.5	7.5	37.6
(2 : 1 : 2)		Light Brown, 60		(32.5)	(2.4)	(5.8)	(13.4)	(7.4)	(37.4)
Cp_2_TiCl_2_ + MThBH_2_ + *n*-BuNH_2_	20	[{Cp_2_TiCl}_2_MThB]	715.46	47.0	3.9	7.8	17.9	9.9	13.4
(2 : 1 : 2)		Light Brown, 65	(715)	(46.8)	(3.7)	(7.6)	(17.5)	(9.6)	(13.1)
Cp_2_ZrCl_2_ + MThBH_2_ + *n*-BuNH_2_	22	[{Cp_2_ZrCl}_2_MThB]	802.14	41.9	3.5	6.9	15.9	8.8	22.7
(2 : 1 : 2)		Cream, 65	(802)	(41.6)	(3.3)	(6.9)	(15.8)	(8.5)	(22.2)
Cp_2_HfCl_2_ + MThBH_2_ + *n*-BuNH_2_	26	[{Cp_2_HfCl}_2_MThB]	976.68	34.4	2.9	5.7	13.1	7.3	36.5
(2 : 1 : 2)		Brown, 60	(977)	(34.2)	(2.8)	(5.6)	(13.0)	(7.2)	(36.4)

**Table 2:** Significant infrared spectral bands (cm^−1^).

Compound	*ν* (C=N)	*ν* (NH)/ *ν* (C−O−C)/ *ν* (C−S−C)	*ν*(M−N)	*ν*(M−S)	*η* ^5^-C_5_H_5_
[{Cp_2_TiCl}_2_MTP]	1580 s, 1560 s	3150 m	440 m	370 m	3000 m, 1430 m, 1020 m
[{Cp_2_ZrCl}_2_MTP]	1585 s, 1560 s	3140 m	435 m	360 m	3010 m, 1420 m, 1025 m
[{Cp_2_HfCl}_2_MTP]	1578 s, 1555 s	3145 m	420 m	340 w	3010 m, 1430 m, 1020 w
[{Cp_2_TiCl}_2_MTE]	1570 s, 1550 s	3155 m	435 m	360 m	3015 m, 1425 m, 1015 w
[{Cp_2_ZrCl}_2_MTE]	1575 s, 1550 s	3150 m	430 m	340 m	3000 m, 1435 m, 1020 m
[{Cp_2_HfCl}_2_MTE]	1570 s, 1545 s	3150 m	425 m	340 m	3005 m, 1425 m, 1020 w
[{Cp_2_TiCl}_2_MTB]	1560 s, 1540 s	3145 m	440 m	355 m	3000 m, 1430 m, 1030 m
[{Cp_2_ZrCl}_2_MTB]	1560 s, 1545 s	3140 m	435 m	345 m	3005 m, 1425 m, 1020 m
[{Cp_2_HfCl}_2_MTB]	1555 s, 1540 s	3140 m	425 m	340 m	3000 m, 1425 m, 1025 w
[{Cp_2_TiCl}_2_MOP]	1565 s, 1545s	1350 m, 1290 m	430 m	360 m	3000 w, 1430m, 1020 m
[{Cp_2_ZrCl}_2_MOP]	1560 s, 1540 s	1345 m, 1290 m	425 m	340 m	2990 w, 1420 m, 1015m
[{Cp_2_HfCl}_2_MOP]	1570 s, 1550 s	1350 m, 1280 m	420 m	340 m	2980 w, 1420 m, 1020 w
[{Cp_2_TiCl}_2_MOE]	1578 s, 1555 s	1355 m, 1285 m	425 m	355 m	3015 w, 1425 m, 1025 w
[{Cp_2_ZrCl}_2_MOE]	1575 s, 1550 s	1350 m, 1280 m	420 m	350 m	3010 w, 1420 m, 1020 m
[{Cp_2_HfCl}_2_MOE]	1570 s, 1550 s	1340 m, 1285 m	410 m	340 m	3000 m, 1415 m, 1015w
[{Cp_2_TiCl}_2_MOB]	1575 s, 1555 s	1355 m, 1280 m	430 m	375 m	3000 w, 1430 m, 1025 m
[{Cp_2_ZrCl}_2_MOB]	1570 s, 1550s	1350 m, 1285 m	428 m	360 m	3000 w, 1420 m, 1020m
[{Cp_2_HfCl}_2_MOB]	1580 s, 1555 s	1345 m, 1280 m	420 m	360 m	3000 m, 1430 m, 1010 w
[{Cp_2_TiCl}_2_MThP]	1570 s, 1550 s	660 m	430 m	375 m	3000 w, 1420 m, 1020m
[{Cp_2_ZrCl}_2_MThP]	1565 s, 1540 s	650 m	425 m	370 m	3000 w, 1425 m, 1010 m
[{Cp_2_HfCl}_2_MThP]	1565 s, 1545 s	655 m	415 m	360 m	2980 w, 1425 m, 1020 w
[{Cp_2_TiCl}_2_MThE]	1560 s, 1540 s	650 m	425 m	380 m	3000 w, 1430 m, 1020 m
[{Cp_2_ZrCl}_2_MThE]	1565 s, 1545 s	655 m	420 m	370 m	2995 w, 1420 m, 1025m
[{Cp_2_HfCl}_2_MThE]	1560 s, 1545 s	650 m	410 m	365 m	2990 w, 1420 m, 1010 w
[{Cp_2_TiCl}_2_MThB]	1570 s, 1555 s	660 m	435 m	375 m	3005 w, 1425 m, 1010 m
[{Cp_2_ZrCl}_2_MThB]	1575 s, 1550 s	650 m	430 m	370 m	3000 w, 1420 m, 1015 m
[{Cp_2_HfCl}_2_MThB]	1580 s, 1560 s	655 m	410 m	360 m	2985 w, 1430 m, 1015 w

**Table 3:** Significant NMR data *δ*, ppm).

Compound	^1^H NMR				^13^C NMR			
	*η* ^5^-C_5_H_5_	−NH−	−CH_2_−	Phenyl ring	*η* ^5^-C_5_H_5_	R	−N=C−S	−R−C=N
[{Cp_2_TiCl}_2_MTP]	6.60 s	9.50 s	-	7.20–7.35 m	115.8	135.2, 130.6, 128.5	178.2	163.2
[{Cp_2_ZrCl}_2_MTP]	6.55 s	9.40 s	-	7.25–7.38 m	115.6	132.5, 130.2, 127.4	177.5	163.0
[{Cp_2_HfCl}_2_MTP]	6.60 s	9.52 s	-	7.30–7.45 m	115.3	134.1, 130.4, 128.6	176.2	162.8
[{Cp_2_TiCl}_2_MTE]	6.52 s	9.48 s	2.45 s	-	115.7	24.8	178.3	166.0
[{Cp_2_ZrCl}_2_MTE]	6.58 s	9.45 s	2.50 s	-	115.4	24.6	177.6	165.6
[{Cp_2_HfCl}_2_MTE]	6.58 s	9.42 s	2.48 s	-	115.1	24.5	176.2	165.0
[{Cp_2_TiCl}_2_MTB]	6.70 s	9.50 s	1.80–2.20 m	-	115.8	22.8, 14.6	181.2	167.9
[{Cp_2_ZrCl}_2_MTB]	6.72 s	9.48 s	1.90–2.25 m	-	115.4	21.6, 14.0	178.8	167.0
[{Cp_2_HfCl}_2_MTB]	6.65 s	9.40 s	1.80–2.18 m	-	115.2	21.8, 14.0	177.9	165.8
[{Cp_2_TiCl}_2_MOP]	6.58 s	-	-	7.32–7.48 m	115.7	140.2, 135.6, 132.6	180.2	165.8
[{Cp_2_ZrCl}_2_MOP]	6.60 s	-	-	7.30–7.50 m	115.5	138.5, 134.2, 130.5	178.8	165.4
[{Cp_2_HfCl}_2_MOP]	6.75 s	-	-	7.38–7.50 m	115.1	138.0, 134.4, 130.6	177.6	165.0
[{Cp_2_TiCl}_2_MOE]	6.62 s	-	2.50 s	-	115.8	26.5	180.8	167.0
[{Cp_2_ZrCl}_2_MOE]	6.65 s	-	2.52 s	-	115.4	25.7	179.0	166.8
[{Cp_2_HfCl}_2_MOE]	6.72 s	-	2.48 s	-	115.2	25.0	177.9	166.2
[{Cp_2_TiCl}_2_MOB]	6.72 s	-	2.0–2.20 m	-	115.4	24.6, 16.2	182.4	169.2
[{Cp_2_ZrCl}_2_MOB]	6.72 s	-	1.92–2.18 m	-	115.3	23.7, 15.8	179.2	168.4
[{Cp_2_HfCl}_2_MOB]	6.70 s	-	1.90–2.16 m	-	115.1	23.0, 15.9	178.2	167.2
[{Cp_2_TiCl}_2_MThP]	6.62 s	-	-	7.40–7.52 m	115.3	133.2, 130.0, 127.5	177.0	162.1
[{Cp_2_ZrCl}_2_MThP]	6.58 s	-	-	7.35–7.50 m	115.1	130.4, 129.3, 126.2	176.5	161.8
[{Cp_2_HfCl}_2_MThP]	6.70 s	-	-	7.35–7.48 m	115.0	133.1, 128.8, 126.6	174.2	161.6
[{Cp_2_TiCl}_2_MThE]	6.65 s	-	2.40 s	-	115.4	23.7	176.1	164.7
[{Cp_2_ZrCl}_2_MThE]	6.68 s	-	2.35 s	-	115.3	22.6	175.7	163.2
[{Cp_2_HfCl}_2_MThE]	6.60 s	-	2.32 s	-	115.0	22.1	173.0	162.8
[{Cp_2_TiCl}_2_MThB]	6.70 s	-	1.8–2.0 m	-	115.6	21.6, 13.9	180.7	165.8
[{Cp_2_ZrCl}_2_MThB]	6.72 s	-	1.92–2.15 m	-	115.3	20.4, 13.6	177.1	166.0
[{Cp_2_HfCl}_2_MThB]	6.68 s	-	1.92–2.20 m	-	115.2	20.1, 13.0	175.0	164.6

**Table 4:** Antifungal activity of bis(mercaptoazoles) and their titanium(IV)/zirconium(IV)/hafnium(IV) complexes.

Compound	Average % inhibition after 96 h								
	*A. niger*			*A. alternata*			*H. oryzae*		
	1000	100	10	1000	100	10	1000	100	10
MTPH_2_	44.8	32.7	25.2	45.0	32.8	22.8	42.8	26.2	18.5
[{Cp_2_TiCl}_2_MTP]	70.8	58.4	42.8	70.0	59.2	37.6	66.8	52.7	43.2
[{Cp_2_ZrCl}_2_MTP]	65.8	48.2	40.2	61.8	46.2	32.7	55.2	47.6	32.8
[{Cp_2_HfCl}_2_MTP]	65.0	46.7	40.0	61.9	44.8	30.2	54.8	42.8	30.6
MTEH_2_	35.8	26.2	20.8	38.0	24.8	18.8	33.6	22.6	15.8
[{Cp_2_TiCl}_2_MTE]	64.8	52.6	32.8	60.7	50.3	32.8	58.8	42.4	33.8
[{Cp_2_ZrCl}_2_MTE]	60.2	44.8	30.7	54.2	32.8	29.7	42.7	40.0	25.8
[{Cp_2_HfCl}_2_MTE]	59.6	40.2	30.0	54.0	30.6	22.8	42.0	39.6	24.8
MTBH_2_	40.6	30.8	24.2	40.2	30.6	21.6	40.6	25.5	17.2
[{Cp_2_TiCl}_2_MTB]	68.2	55.6	40.3	65.8	52.8	36.2	60.1	47.2	40.1
[{Cp_2_ZrCl}_2_MTB]	64.8	46.1	35.2	58.9	40.7	30.8	50.4	46.2	30.6
[{Cp_2_HfCl}_2_MTB]	64.1	42.2	32.7	54.8	39.2	28.2	48.8	44.1	28.5
MOPH_2_	32.4	24.8	18.8	30.0	22.6	16.2	28.2	20.5	15.1
[{Cp_2_TiCl}_2_MOP]	64.2	50.8	28.4	58.2	44.8	27.6	56.6	42.0	29.8
[{Cp_2_ZrCl}_2_MOP]	56.0	40.0	26.2	50.8	28.6	20.2	40.6	30.8	21.6
[{Cp_2_HfCl}_2_MOP]	54.2	38.6	25.8	50.0	26.2	20.0	38.8	28.6	20.8
MOEH_2_	25.8	20.2	15.8	26.2	18.5	14.4	22.8	16.0	12.7
[{Cp_2_TiCl}_2_MOE]	56.3	42.6	22.8	52.8	36.2	21.8	48.2	36.8	21.7
[{Cp_2_ZrCl}_2_MOE]	50.5	32.0	18.5	47.8	21.2	14.8	32.6	20.8	14.8
[{Cp_2_HfCl}_2_MOE]	50.0	31.8	16.2	46.2	21.8	14.0	33.8	18.9	14.5
MOBH_2_	28.9	22.3	16.8	29.8	20.5	15.8	26.0	18.2	14.6
[{Cp_2_TiCl}_2_MOB]	60.0	48.2	24.6	54.0	40.6	24.2	52.8	40.2	26.8
[{Cp_2_ZrCl}_2_MOB]	55.2	38.6	22.8	49.2	24.2	16.3	38.2	24.0	16.5
[{Cp_2_HfCl}_2_MOB]	54.0	36.8	21.7	48.6	24.0	15.3	36.8	22.8	16.0
MThPH_2_	56.8	40.2	31.8	55.6	42.8	30.6	52.5	38.8	28.5
[{Cp_2_TiCl}_2_MThP]	85.6	74.8	53.2	80.7	68.2	50.8	78.4	66.8	52.8
[{Cp_2_ZrCl}_2_MThP]	78.2	60.5	50.8	76.2	58.6	48.2	70.5	60.2	46.7
[{Cp_2_HfCl}_2_MThP]	76.0	60.1	48.2	72.8	56.2	48.0	70.2	58.8	45.8
MThEH_2_	48.2	35.6	26.8	45.2	35.8	24.1	44.6	27.8	20.2
[{Cp_2_TiCl}_2_MThE]	75.4	62.3	44.6	70.2	60.6	40.5	68.2	55.2	46.8
[{Cp_2_ZrCl}_2_MThE]	67.2	50.1	42.6	63.8	50.1	34.8	59.1	50.2	38.8
[{Cp_2_HfCl}_2_MThE]	67.0	49.8	41.8	62.9	49.9	32.7	57.2	50.1	36.2
MThBH_2_	50.6	38.2	29.6	50.2	37.6	26.2	48.8	30.6	24.2
[{Cp_2_TiCl}_2_MThB]	78.2	68.5	50.2	76.0	60.8	42.8	70.6	60.5	50.4
[{Cp_2_ZrCl}_2_MThB]	70.8	54.2	45.8	68.2	54.2	40.5	62.3	52.8	40.7
[{Cp_2_HfCl}_2_MThB]	69.7	52.8	42.7	67.8	52.0	38.2	65.8	54.2	40.8

**Table 5:** Antibacterial activity of titanium(IV)/zirconium(IV)/hafnium(V) complexes with bis(mercaptoazoles).

Compound	Diameter of inhibition zone (mm)	
	*B. subtilis*	*E. coli*
[{Cp_2_TiCl}_2_MTP]	16	14
[{Cp_2_ZrCl}_2_MTP]	15	13
[{Cp_2_HfCl}_2_MTP]	10	11
[{Cp_2_TiCl}_2_MTE]	15	13
[{Cp_2_ZrCl}_2_MTE]	14	11
[{Cp_2_HfCl}_2_MTE]	14	10
[{Cp_2_TiCl}_2_MTB]	14	14
[{Cp_2_ZrCl}_2_MTB]	13	12
[{Cp_2_HfCl}_2_MTB]	10	8
[{Cp_2_TiCl}_2_MOP]	14	13
[{Cp_2_ZrCl}_2_MOP]	12	11
[{Cp_2_HfCl}_2_MOP]	10	12
[{Cp_2_TiCl}_2_MOE]	13	12
[{Cp_2_ZrCl}_2_MOE]	11	10
[{Cp_2_HfCl}_2_MOE]	8	11
[{Cp_2_TiCl}_2_MOB]	13	12
[{Cp_2_ZrCl}_2_MOB]	11	10
[{Cp_2_HfCl}_2_MOB]	7	9
[{Cp_2_TiCl}_2_MThP]	20	18
[{Cp_2_ZrCl}_2_MThP]	17	16
[{Cp_2_HfCl}_2_MThP]	18	15
[{Cp_2_TiCl}_2_MThE]	18	17
[{Cp_2_ZrCl}_2_MThE]	16	16
[{Cp_2_HfCl}_2_MThE]	15	14
[{Cp_2_TiCl}_2_MThB]	17	15
[{Cp_2_ZrCl}_2_MThB]	15	15
[{Cp_2_HfCl}_2_MThB]	13	12

## References

[1] Raper ES (1996). Complexes of heterocyclic thionates—part 1: complexes of monodentate and chelating ligands. *Coordination Chemistry Reviews*.

[2] Raper ES (1994). Copper complexes of heterocyclic thioamides and related ligands. *Coordination Chemistry Reviews*.

[3] Raper ES (1985). Complexes of heterocyclic thione donors. *Coordination Chemistry Reviews*.

[4] Al-Obaidi KH, Ali BF, Abu-El-Halawa R, Abo-Amer A (2004). Synthesis of 1,3,4-mercapto-oxadiazole mono- and dinuclear copper(I) and copper(II) complexes and their microbiological activity. *Transition Metal Chemistry*.

[5] Ma C, Jiang Q, Zhang R (2004). Syntheses, characterizations and crystal structures of new organotin complexes with 2-mercapto-6-nitrobenzothiazole. *Polyhedron*.

[6] Bell NA, Clegg W, Creighton JR, Raper ES (2000). Complexes of heterocyclic thiones and group 12 metals—part 2: the chemical and 
electrochemical synthesis of mercury(II) complexes of 1-methylimidazoline-2(3H)-thionate. The crystal structure of *trans*-[bis–{(*η*
^1^–*S*–1–methylimidazoline-2(3H)-thionate)(*η*
^1^–*S*–1–methylimidazoline line-2(3H)-thionate)-(μ_2_-*S, N*-1-methylimidazoline-2-thionate)mercury(II)}] at 160K. *Inorganica Chimica Acta*.

[7] Yadav S, Pandey OP, Sengupta SK (1995). Synthesis, physico-chemical and biological studies on oxovanadium(IV) derivatives of mercaptotriazoles. *Transition Metal Chemistry*.

[8] Oik R-M, Oik B, Dietzsch W, Kirmse R, Hoyer E (1992). The chemistry of 1,3-dithiole-2-thione-4,5-dithiolate (dmit). *Coordination Chemistry Reviews*.

[9] Blower PJ, Dilworth JR (1987). Thiolato-complexes of the transition metals. *Coordination Chemistry Reviews*.

[10] Jaiswal AK, Rao GP, Pandey OP, Sengupta SK (1998). Efficacy of organophosphorus derivatives against fungal pathogens of sugarcane. *Journal of Agricultural and Food Chemistry*.

[11] Pandey OP, Sengupta SK, Mishra MK, Tripathi CM (2003). Synthesis, spectral and antibacterial studies of binuclear titanium(IV) / zirconium(IV) complexes of piperazine dithiosemicarbazones. *Bioinorganic Chemistry and Applications*.

[12] Maruzzella JC, Henry PA (1958). The antimicrobial activity of perfume oils. *Journal of the American Pharmaceutical Association*.

[13] Pandey OP, Sengupta SK, Tripathi CM (2005). Reactions of Cp_2_MCI_2_ (M=Ti or Zr) with imine-oxime ligands. Formation of metallacycles. *Molecules*.

[14] Ibrahim KM, Mostafa SI, Nawar N, Younis ZA (2004). Synthesis and structure studies of Co(II), Ni(II), Cu(II), Pd(II), Ru(II), Ag(I), Cd(II) and dioxouranium(VI) complexes with 1-acetoacet-o-toluidide-4- phenyl-3-thiosemicarbazone. *Indian Journal of Chemistry, Section A*.

[15] Goel S, Pandey OP, Sengupta SK (1989). Synthesis and physico-chemical studies on neodymium(III) and samarium(III) derivatives with mercapto triazoles. *Bulletin de la Société Chimique de France*.

[16] Sengupta SK, Pandey OP, Rai A, Sinha A (2000). Lanthanum(III) and praseodymium(III) complexes of acetylferrocenyl mercaptotriazoles. *Transition Metal Chemistry*.

[17] Sengupta SK, Pandey OP, Srivastava AK, Rai R, Mishra KD (1999). Mono- and bis(cyclopentadienyl)titanium(IV)/zirconium(IV) derivatives with substituted mercapto triazines. *Indian Journal of Chemistry, Section A*.

[18] Srivastava BK, Srivastava SK, Pandey OP, Sengupta SK (1997). Synthesis and spectroscopic studies of mono-(cyclopentadienyl)titanium(IV) zirconium(IV) 
and bis(cyclopentadienyl)titanium(IV)/zirconium(IV) derivatives with acetylferrocenylmercaptotriazoles. *Gazzetta Chimica Italiana*.

[19] Wailes PC, Coutts RSP, Weigold H (1974). *Organometallic Chemistry of Titanium, Zirconium and Hafnium*.

[20] Nakamoto K (1997). *Infrared and Raman Spectra of Inorganic and Coordination Compounds*.

[21] Carcelli M, Mazza P, Pelizzi C, Pelizzi G, Zani F (1995). Antimicrobial and genotoxic activity of 2,6-diacetylpyridine bis(acylhydrazones) and their complexes with some first transition series metal ions. X-ray crystal structure of a dinuclear copper(II) complex. *Journal of Inorgnic Biochemistry*.

